# Association of Medical Superintendents of American Institutions for the Insane

**Published:** 1846-10

**Authors:** 


3. *Association of Medical Superintendents of American Institutions for the Insane.*—The Association of Medical Superintendents of American Institutions for the Insane, held its second meeting at Coleman's Hotel, in the City of Washington, on the 11th of May, 1846, the President, Samuel B. Woodward, M. D., in the chair, and Thomas S. Kirkbride, M. D., Secretary.

*Present.*

Dr. Samuel B. Woodward, of the Massachusetts State Lunatic Hospital at Worcester.

Dr. James Bates, of the Maine Insane Hospital, Augusta.

Dr. Andrew McFarland, of the New Hampshire State Hospital at Concord.

Dr. William H. Rockwell, of the Vermont State Hospital at Brattleborough.

Dr. Luther N. Bell, of the McLean Asylum for the Insane, at Somerville, Mass.

Dr. C. H. Stedman, of the Boston Lunatic Asylum.

Dr. N. Cutler, of the Pepperell Asylum, Mass.

Dr. George Chandler, of the Massachusetts State Lunatic Hospital, at Worcester.

Dr. John S. Butler, of the Connecticut Retreat at Hartford.

Dr. Amariah Brigham, of the New York State Lunatic Asylum at Utica.

Dr. Pliny Earle, of the Bloomingdale Asylum, New York.

Dr. G. H. White of the Hudson Lunatic Asylum, New York.

Dr. James Macdonald, of the Private Institution, Flushing, New York.

Dr. Thomas S. Kirkbride, of the Pennsylvania Hospital for the Insane, Philadelphia.

Drs. Stewart and Fenerden of the Maryland Hospital.

Dr. I. P. Stokes, of the Mount Hope Asylum at Baltimore.

Dr. Wm. M. Awl, of the Ohio State Hospital, at Columbus.

Dr. John M. Galt, of the Eastern Asylum of Virginia, at Williamsburgh.

Dr. I. W. Parker, of the South Carolina Hospital, at Columbia.

Dr. Walter Tellfer, of the Lunatic Hospital, at Toronto, Canada.

Dr. Wm. M. Awl, of the Ohio State Hospital, was elected Vice President of the Association, in the place of Dr. Samuel White, of Hudson, deceased.

Reports were received and read from various committees appointed at the last meeting of the Association:—

On the subject of the Moral Treatment of Insanity, by Dr. Amariah Brigham; on the Medical Treatment of Insanity, by Dr. Samuel B. Woodward; on Restraint and Restraining Apparatus, by Dr. Bell; on the Construction of Hospitals for the Insane, by Dr. Awl; on the Jurisprudence of Insanity, by Dr. Ray; on the Organization of Hospitals for the Insane, by Dr. Kirkbride; on the Statistics of Insanity, by Dr. Earle; on Asylums for Idiots and the Demented, by Dr. Brigham; on Chapels and Chaplains in Insane Hospitals, by Dr. Butler; on Post Mortem Examinations, by Dr. Kirkbride; on Asylums for Colored Persons, by Dr. Galt; on the proper provision for Insane Prisoners, by Dr. Brigham. An Essay on the Construction of Hospitals for the Insane was also read by Dr. Bell, and the subjects embraced in the Reports were minutely discussed by the Members of the Association.

The following preamble and resolutions were adopted:—

*Whereas*, Since the last meeting of this Association, Dr. Samuel White of New York, the venerable and highly respected late Vice-President of this Association, has died:—

Therefore,

*Resolved*, That by the death of Dr. White, the Association, and the Medical Profession, have lost an esteemed and valued member, and the cause of humanity a useful and active friend. Particularly have the friends of the insane reason to mourn his loss, as he had long been successfully engaged in relieving the sufferings of this afflicted class of his fellow beings, and, by his labors and his writings, essentially aided in improving their condition.

*Resolved*, That we deeply sympathise with the surviving members of his family, and recall, at the present time, the excellencies of his character, his useful precepts, and the worthy example he presented of a Gentleman, Physician, and Christian, devoted to deeds of goodness, and whose long and active life was spent in promoting the welfare of his fellow men.

*Resolved*, That Dr. Brigham be requested to prepare an obituary notice of the late Dr. White, to be entered upon the minutes of the Association, and to be published.

*Resolved*, That the Secretary of this Association present a copy of these resolutions to the nearest relative of the deceased.

The following resolutions were also adopted by the Association during its different sessions:

*Resolved*, That the resolution of the last meeting, relative to Members of the Association, be so amended as to read as follows:—That the Medical Superintendents of the various incorporated, or other legally constituted Institutions for the Insane, now existing on this continent, or which may be commenced prior to next meeting, and all those who have heretofore been Medical Superintendents and Members of this Association, or who may be hereafter appointed to these stations, be, and they are hereby constituted Members of the Association.

*Resolved*, That in future, each regularly constituted Institution for the Insane on this continent, may have one representative in this Association;—that as heretofore, this shall be the Medical Superintendent, where such officer exists, but in those institutions in which there is a different organization, it may be either of the regular medical officers who may find it most convenient to attend.

*Resolved*, That the subjects, upon which committees were appointed at the first meeting of the Association, be continued, each in the hands of the Chairman of the respective Committees, to be reported upon at the next meeting.

*Resolved*, That in addition, each one of the following subjects be confided to a single Member of the Association, who is hereby requested to report at the next meeting of the Association:—

1. Treatment of Incurables. Dr. Macdonald.

2. Is there any relation between Phrenology and Insanity? Dr. Fenerden.

3. The Classification of Insanity. Dr. Earle.

4. The admission of visitors into the halls of the patients. Dr. Ray.

5. Visits to, and correspondence with patients by their friends. Dr. Stokes.

6. The comparative value of the different kinds of manual labor for patients, and the best means of employment in winter. Dr. Rockwell.

7. The proper number of patients for one institution. Dr. Brigham.

8. The utility of night attendants, and the propriety of not locking patients' doors during the night. Dr. Chandler.

9. The advantages and disadvantages of cottages, for wealthy patients, adjacent to Hospitals for the Insane. Dr. Kirkbride.

10. The relative value of the different kinds of fuel for heating hospitals. Dr. Bates.

11. Insanity, and the condition of the insane in the British Provinces. Dr. Tellfer.

12. The nature and treatment of Insanity produced by the use of intoxicating liquors. Dr. Stedman.

13. The relations of Menstruation to Insanity. Dr. Fenerden.

14. Under what circumstances can the Insane of the poorer classes be properly treated with the greatest degree of economy. Dr. McFarland.

15. The effects upon the Insane of the use of Tobacco. Dr. Cutler.

16. Reading, recreation and amusement for the Insane. Dr. Galt.

17. On water closets in the wards and yards of Hospitals for the Insane. Dr. Bell.

18. On the construction and arrangement of Institutions for the Insane in Southern climates. Dr. Parker.

*Resolved,* That the members of this Association be urgently requested, with the concurrence of the friends of patients, to make post mortem examinations, in all cases of insanity which may prove fatal while under their care, and to report the result of their observations at the next meeting of the Association.

*Resolved,* That each Member of this Association be requested to ascertain the facts and circumstance (such as sex, age, civil state, vocation, mode and other matters susceptible of being tabularized,) of each case of suicide, occurring in his respective State, between the first day of January and the last day of December, 1847, and forward an abstract of the same as soon after the latter date as convenient, to the Chairman of the Committee on Suicide: it being understood that in States having more than one Member, they be requested to divide their States by certain territorial limits.

*Resolved,* That it be recommended to the officers of the different Institutions for the Insane in this country, to have engraved, previous to the next meeting of the Association, a view and ground plan of their respective establishments, and of a size that will permit their being bound with their Annual Reports.

*Resolved,* That a committee of three be appointed to publish, in a collected form, the Transactions of the Association, or, under certain circumstances, such parts of the same as they may deem expedient.

*Resolved,* That the essays presented to this Association are understood to be the opinions of the Chairmen of the different Committees by whom they have been reported, and do not necessarily express the sentiments of other members relative to their details.

*Resolved,* That in case the Committee do not publish any of the essays, their writers then have the privilege of publishing them in a separate form, should they deem it expedient to do so.

*Resolved,* That the Secretary be directed to publish an abstract of the Proceedings of the Association, in the American Journal of Insanity, the American Journal of the Medical Sciences, and the New York Journal of Medicine.

The Association continued its session till the evening of the 14th of May, and adjourned to meet in the city of New York on the 2d Monday of May, 1848, at 10 A. M.

By order of the Association,
THOMAS S. KIRKBRIDE, *Secretary.*

This Association, first instituted during the last year, evinces a determination on the part of its members, that no efforts on their part shall be wanting to enable the Department, peculiarly their own, to keep pace with the advancement of other branches of Medical Science. From the well earned reputation of those composing the Association, and the responsible stations they hold, together with the vast field for observation afforded by the Institutions under their control, we look upon it as destined to do much to ameliorate the condition and improve the treatment of the unfortunate class of sufferers for whose benefit it was instituted. We are indebted to the *New York Journal of Medicine*, for the above abstract of the proceedings of the second meeting.

